# Long-term comparison of renal and metabolic outcomes after sodium–glucose co-transporter 2 inhibitor or glucagon-like peptide-1 receptor agonist therapy in type 2 diabetes

**DOI:** 10.1186/s12916-024-03483-z

**Published:** 2024-07-02

**Authors:** Minji Sohn, Seoungyeon Nam, Michael A. Nauck, Soo Lim

**Affiliations:** 1 Department of Internal Medicine, Seoul National University Bundang Hospital, Seoul National University College of Medicine, 82, Gumi-ro 173 Beon-gil, Bundang-gu, Seongnam-City, 13620 South Korea; 2 Section Diabetes, Endocrinology, Metabolism, Medical Department I Katholisches Klinikum Bochum gGmbH, St. Josef Hospital Ruhr-University Bochum, Bochum, Germany

**Keywords:** SGLT2 inhibitor, GLP1 receptor agonist, Renal outcome, eGFR, Albuminuria

## Abstract

**Background:**

Renal outcomes in patients with type 2 diabetes following treatment with sodium–glucose co-transporter-2 inhibitors (SGLT2is) or glucagon-like peptide-1 receptor agonists (GLP1RAs) have not been directly compared. This study compared the impact of SGLT2i and GLP1RA therapy on renal function and metabolic parameters.

**Methods:**

Patients with type 2 diabetes who initiated SGLT2i or GLP1RA therapy in a tertiary hospital between January 2009 and August 2023 were included to assess composite renal outcomes, such as a 40% decline in estimated glomerular filtration rate (eGFR), onset of end-stage renal disease, renal death, or new-onset macroalbuminuria. Alterations in blood pressure, glucose regulation parameters, lipid profile, and anthropometric parameters, including body fat and muscle masses, were examined over 4-years.

**Results:**

A total of 2,112 patients were enrolled using a one-to-three propensity-score matching approach (528 patients for GLP1RAs, 1,584 patients for SGLT2i). SGLT2i treatment was favoured over GLP1RA treatment, though not significantly, for composite renal outcomes (hazard ratio [HR], 0.63; *p* = 0.097). SGLT2i therapy preserved renal function effectively than GLP1RAs (decrease in eGFR, ≥ 40%; HR, 0.46; *p* = 0.023), with improving albuminuria regression (HR, 1.72; *p* = 0.036). SGLT2i therapy decreased blood pressure and body weight to a greater extent. However, more patients attained HbA_1c_ levels < 7.0% with GLP1RAs than with SGLT2is (40.6% vs 31.4%; *p* < 0.001). GLP1RA therapy enhanced β-cell function and decreased LDL-cholesterol levels below baseline values.

**Conclusions:**

SGLT2is were superior for preserving renal function and reducing body weight, whereas GLP1RAs were better for managing glucose dysregulation and dyslipidaemia.

**Supplementary Information:**

The online version contains supplementary material available at 10.1186/s12916-024-03483-z.

## Background

Type 2 diabetes is a multifactorial, chronic metabolic disorder that afflicts more than 600 million people worldwide, often leading to severe complications, including cardiovascular and renal diseases [1]. The therapeutic management of this complex disease provides an ongoing clinical challenge, necessitating the development of a diverse spectrum of pharmacological agents targeting various pathophysiological aspects. Among these, sodium–glucose co-transporter 2 inhibitors (SGLT2is) and glucagon-like peptide-1 receptor agonists (GLP1RAs) have emerged as two promising antidiabetic agents. Both have demonstrated efficacy in glycaemic control, cardiovascular risk mitigation, and improvement of renal outcomes in placebo-controlled randomized clinical trials (RCTs) [2, 3].

In addition to cardiovascular diseases (CVDs), diabetic nephropathy poses a significant clinical burden in type 2 diabetes and is a leading cause of mortality in affected patients [4]. Both SGLT2is and GLP1RAs have demonstrated favourable impacts on renal outcomes in RCTs [5, 6]. To the best of our knowledge, however, the impact of these two medication classes on renal outcomes has not yet been compared directly. Although there have been some meta-analyses comparing these effects [3, 7], such indirect comparisons are limited by inconsistent definitions of composite renal outcomes across the studies [8]. For example, several GLP1RA trials have included new-onset macroalbuminuria for defining composite renal outcomes [9, 10]. Conversely, SGLT2i trials have adopted different criteria for the albuminuria component. For example, development of albuminuria, regression from albuminuria to normal, or progression to overt proteinuria were used in respective studies [3, 11, 12]. Moreover, the criteria for diminished renal function have varied, with some trials depending on a percent decrease in estimated glomerular filtration rate (eGFR) [13] and others using a doubling of serum creatinine [14].

Few studies have specifically assessed the long-term differential impacts of SGLT2i and GLP1RA therapies on renal outcomes and related metabolic markers [15]. Our recent observations have revealed a potential association between renal function parameters, such as eGFR and albuminuria, and the variability in the effectiveness of these agents in averting cardiovascular events, thereby highlighting the crucial importance of renal management [5]. Definitive conclusions regarding renal outcomes have not been reached due to the limited number of studies designating them as primary endpoints. Against this background, the present study aimed to employ a real-world data approach to comprehensively compare the renal and metabolic consequences associated with SGLT2i and GLP1RA therapy in patients with type 2 diabetes.

## Methods

### Study design and population

We used a propensity-score matched cohort design that was approved by an independent Ethics Committee/Institutional Review Board (B-2103–675-103), with a waiver for patient-informed consent due to its retrospective nature. We included data from adult patients with type 2 diabetes who attended the diabetes clinic at Seoul National University Bundang Hospital, South Korea, between January 2009 and August 2023 and who satisfied the following inclusion criteria: (1) aged 19 years or older; (2) receiving a new prescription of SGLT2i or GLP1RA for a minimum of 90 days; and (3) with a documented baseline albuminuria status. Patients with medication adherence less than 70% to SGLT2i or GLP1RA therapy, or those who used both agents concomitantly, were excluded.

Over an up-to 4-year observation period, commencing from the date of prescription, evaluations were conducted at predefined intervals of 180, 365, 540, 730, 1,095, and 1,460 days. In addition, an exhaustive data collection was performed throughout this period to assess trends in clinical parameters. This investigation conformed to the STROBE statement for cohort studies.

### Outcomes

The primary endpoint of this study comprised composite renal outcomes, which included a sustained reduction in eGFR of ≥ 40%, end-stage renal disease (ESRD), newly confirmed macroalbuminuria (evaluated by urinary albumin-to-creatinine ratio [ACR] assessment), and kidney-related mortality. Secondary outcomes comprised the risk assessment for each component of the primary outcome and the regression of albuminuria. Additionally, fractional excretion of glucose (FE_glc_), sodium (FE_Na_), and potassium (FE_K_) were measured. Glycaemic control parameters (glycated haemoglobin [HbA_1c_], insulin, and glucagon), liver enzyme activities, and lipid profiles were also checked. Anthropometric parameters including body weight and blood pressure were monitored systematically. The specific definitions of these outcomes are detailed in Additional file 1: Table S1.

### Data collection and measurements

Clinical data, including outpatient care details, admission records, laboratory values, anthropometric assessments, and prescription information, were extracted from the clinical database. Urinary albumin concentration was quantified using turbidimetry (502X; A&T, Tokyo, Japan) and urinary creatinine was evaluated using the Jaffe method (Hitachi 7170; Hitachi, Tokyo, Japan). Albuminuria was identified using the ratio of urinary ACR (mg/g). The eGFR was calculated using the Modification of Diet in Renal Disease (MDRD) equation, which was applied consistently throughout the study.

Composite urinary analyses in the study hospital routinely measure urinary glucose excretion and electrolytes, which enabled us to calculate the urinary fractional excretion of glucose, sodium, and potassium: FE-solute = (urine solute × serum creatinine)/(serum solute × urine creatinine). We calculated medication compliance as the medication possession ratio, defined by the number of days with medication prescribed within the visit time interval.

Anthropometric measurements, such as height and body weight, were collected using standardized protocols. Systolic (SBP) and diastolic blood pressure (DBP) were measured with the participant in a seated position using an electronic blood pressure meter (UA-1020 device; A&D, Tokyo, Japan).

Body composition, including muscle mass and fat mass, was assessed using bioelectrical impedance analysis (InBody720; InBody Co., Seoul, South Korea). Plasma glucose concentrations were ascertained using the glucose oxidase method (747 Clinical Chemistry Analyzer; Hitachi). To estimate pancreatic β-cell function and insulin resistance, the homeostasis model assessment of insulin resistance (HOMA-IR) and β-cell function (HOMA-β) indices were calculated [16]. Plasma HbA_1c_ was measured using a Variant II Turbo HPLC Hemoglobin Testing System (Bio-Rad, Hercules, CA, USA). Total cholesterol, triglycerides, high-density lipoprotein (HDL)-cholesterol, and low-density lipoprotein (LDL)-cholesterol levels were measured using a 747 Clinical Chemistry Analyzer (Hitachi). Any values that were manifestly incorrect, presented in ambiguous ranges, or deemed physically implausible due to typographical errors, were omitted, as described previously [17].

### Safety parameters

Safety assessments involved the monitoring of common adverse events, comprising gastrointestinal discomfort, urinary and genital infections, ketoacidosis, pancreatitis, malignancies, and hypoglycaemia. Hypoglycaemic incidents were recorded based on patient-reported symptoms and plasma glucose levels (< 70 mg/dL). Severe hypoglycaemic events are defined as plasma glucose levels (< 54 mg/dL) or hypoglycaemia requiring third-party assistance. Serious adverse events included death, hospitalization for any reason, and life-threatening events.

### Statistical analysis

All data analyses were conducted using R software (version 4.1.0; The R Foundation for Statistical Computing, Vienna, Austria). Instances where the prescription or the follow-up observation terminated were treated as censored data. Continuous variables were expressed as mean ± standard deviation (SD), whereas categorical variables were delineated as counts and proportions of subjects. To account for differences in baseline characteristics, propensity-score matching was implemented at a 1:3 ratio through the ‘MatchIt’ package, adding a calliper set at 0.2 ratio, incorporating variables such as age, sex, duration of diabetes, body mass index (BMI), SBP, presence of hypertension, presence of dyslipidaemia, and background antidiabetic medications. Standardized mean difference (SMD) with ≤ 0.1 was considered well balanced after matching. Comparing characteristics after matching showed a balance between the groups, indicating a minimal impact on cohort selection.

Operating under the assumption that data were missing completely at random by Little’s test and were < 50% at each visit, a mixed model for repeated measures was used to evaluate continuous variables monitored longitudinally within the treatment groups, which comprised terms for treatment, visit, and the interaction between treatment and visit, with the baseline measurement included as a covariate. The incidence (per 1,000 person-years) for each outcome event in both the SGLT2i and GLP1RA groups was calculated.

Survival was analysed using a Kaplan–Meier method to estimate cumulative event-free survival rates over time, and the Cox proportional hazards regression model was applied to contrast hazard ratios between the treatment groups. Subgroup analyses were conducted stratified by sex (men vs women), age (< 65 years vs ≥ 65 years), eGFR (≥ 60 mL/min/1.72 m^2^ vs < 60 mL/min/1.72 m^2^), and albuminuria status (normoalbuminuria vs micro- and macroalbuminuria). Given that the initiation timelines for GLP1RAs and SGLT2is may differ, we further refined our matching criteria to include the year of medication commencement. This approach is aimed at mitigating any potential biases by aligning the follow-up durations and accounting for variations in comorbid illness patterns over time between the two medications.

Sensitivity analyses were undertaken for individuals who either continued or did not start the prescription of renin–angiotensin system (RAS) blockers, including angiotensin-converting enzyme inhibitors (ACEis) or angiotensin II receptor blockers (ARBs). This approach was designed to address potential bias in the Cox regression model, aiming to mitigate the confounding influence of RAS blocker usage, a factor intrinsically linked to renal outcomes. Furthermore, considering the impact of baseline renal function on the primary outcome, we categorized proteinuria and eGFR according to the Kidney Disease: Improving Global Outcomes (KDIGO) CKD Work Group [18] and used the 'cmprsk' package for a multivariable competing risk regression with the Fine and Gray model to assess subdistribution hazard ratios (sHR) [19].

For parameters exhibiting substantial variability, such as ketone and glucagon levels, missing values were imputed using the last observation carried forward method, and log-transformed values were used for comparative analyses. The threshold for statistical significance was set at a two-sided *p* < 0.05, in accordance with conventional criteria for hypothesis testing.

## Results

### Patient characteristics

Before propensity-score matching, 11,728 patients with type 2 diabetes met the eligibility criteria. After 1:3 propensity-score matching, the analysis included data from 2,112 patients: 528 with GLP1RA therapy and 1,584 with SGLT2i therapy (Additional file 1: Fig. S1). Baseline characteristics were well balanced (Table 1). The mean age of patients in this cohort was 55.9 ± 13.5 years, and the mean BMI was 27.9 ± 4.3 kg/m^2^. Most patients (86.2%) had an eGFR ≥ 60 mL/min/1.73 m^2^, and over half (59.3%) were concurrently prescribed ACEis or ARBs. The most used agents were dapagliflozin (50.7%) or empagliflozin (45.7%) in the SGLT2i group and dulaglutide (65.5%) or liraglutide (26.1%) in the GLP1RA group (Additional file 1: Table S2).

**Table 1:** Baseline characteristics of the included patients before and after propensity-score matching using age, sex, BMI, SBP or hypertension, dyslipidaemia, and background antidiabetic medications

	**Unmatched cohort**			**Matched cohort**		
	**SGLT2i (*n* = 11,155)**	**GLP1RA (*n* = 573)**	SMD*	**SGLT2i (*n* = 1,584)**	**GLP1RA (*n* = 528)**	SMD*
Sex, male	7417 (66.5)	323 (56.4)	0.209*	889 (56.1)	279 (52.8)	0.066
Age, year	62.1 ± 13.5	57.2 ± 13.8	0.358*	56 ± 13.6	55.6 ± 13.1	0.034
Body weight, kg	73.6 ± 14.8	76.4 ± 16.7	0.176*	76 ± 15.7	76.3 ± 16.4	0.019
Body mass index, kg/m^2^	26.8 ± 4.0	28.1 ± 4.8	0.292*	27.8 ± 4.2	28.2 ± 4.7	0.099
Systolic blood pressure, mmHg	136.0 ± 18.6	137.0 ± 17.4	0.055	136.7 ± 17.5	136.3 ± 17	0.023
Diastolic blood pressure, mmHg	78.0 ± 12.4	79.3 ± 12.0	0.103*	78.9 ± 11.9	78.9 ± 11.6	0.002
Duration of diabetes, year	9.2 ± 6.7	10.3 ± 7.7	0.165*	11.2 ± 8.8	10.4 ± 8.4	0.091
Comorbidity						
Hypertension	9,282 (83.2)	446 (77.8)	0.136*	1,249 (78.9)	411 (77.8)	0.025
Dyslipidaemia	9,030 (81.0)	439 (76.6)	0.106*	1,303 (82.3)	434 (82.2)	0.002
Chronic kidney disease	2,464 (22.1)	134 (23.4)	0.031	573 (36.2)	201 (38.1)	0.039
Cardiovascular disease	4,208 (37.7)	148 (25.8)	0.247*	331 (20.9)	107 (20.3)	0.030
Biochemical parameters						
HbA_1c_, %	7.8 ± 1.5	8.3 ± 1.5	0.296*	8.3 ± 1.5	8.2 ± 1.5	0.061
Fasting glucose, mg/dL	156.3 ± 56.3	163.6 ± 65.6	0.119*	164.6 ± 58.7	160.5 ± 59.2	0.070
Total cholesterol, mg/dL	158.5 ± 43.1	158.8 ± 43.4	0.005	160.7 ± 43.3	159.6 ± 40.8	0.028
Triglyceride, mg/dL	152.2 ± 109.7	154.7 ± 98.0	0.023	163.3 ± 109.7	153.6 ± 99.3	0.092
LDL-cholesterol, mg/dL	89.7 ± 33.5	91.1 ± 34.0	0.042	91.9 ± 33.9	91.8 ± 32.1	0.004
HDL-cholesterol, mg/dL	47.4 ± 11.7	47.2 ± 12.5	0.019	46.4 ± 10.7	47.5 ± 12.2	0.096
eGFR, mL/min/1.73 m^2^	82.8 ± 28.7	87.1 ± 31.2	0.143*	90.1 ± 26.0	88.8 ± 26.8	0.047
eGFR <60 mL/min/1.73 m^2^	1729 (21.4)	93 (18.6)	0.072	202 (12.8)	75 (14.5)	0.050
Albuminuria, mg/g	349.8 ± 1,514.9	266.2 ± 1,117.6	0.063	278 ± 1,082.9	260.3 ± 1,060	0.016
Normoalbuminuria	3,297 (55.4)	245 (58.6)	0.064	915 (57.8)	300 (56.8)	0.019
Microalbuminuria	1,058 (17.8)	54 (12.9)	0.135*	234 (14.8)	73 (13.8)	0.027
Macroalbuminuria	1,592 (26.8)	119 (28.5)	0.038	435 (27.5)	155 (29.4)	0.042
Antidiabetic medications	11,153 (100)	562 (98.1)	0.195*	1,584 (100)	528 (100)	0.001
Metformin	9,207 (82.5)	495 (86.4)	0.106*	1,470 (92.8)	478 (90.5)	0.089
Insulin	2,369 (21.2)	268 (46.8)	0.560*	620 (39.1)	212 (40.2)	0.021
Sulfonylurea	3,865 (34.6)	359 (62.7)	0.584*	1,041 (65.7)	337 (63.8)	0.040
Thiazolidinedione	759 (6.8)	63 (11.0)	0.148*	145 (9.2)	54 (10.2)	0.036
Antihypertensive medications	7,347 (65.9)	375 (65.4)	0.009	993 (62.7)	325 (61.6)	0.023
RAS blockers (ARB or ACEi)	7,493 (67.2)	331 (57.8)	0.195*	929 (58.6)	294 (55.7)	0.060
Calcium channel blockers	4,746 (42.5)	254 (44.3)	0.036	570 (36.0)	180 (34.1)	0.040
Beta blockers	2,583 (23.2)	99 (17.3)	0.147*	179 (11.3)	71 (13.4)	0.065
Diuretics	3,496 (31.3)	155 (27.1)	0.094	354 (22.3)	108 (20.5)	0.046
Lipid-lowering agents	8,904 (79.8)	428 (74.7)	0.123*	1,169 (73.8)	391 (74.1)	0.006
Statin	8,811 (79)	426 (74.3)	0.110*	1,138 (71.8)	388 (73.5)	0.037
Fibrate	524 (4.7)	32 (5.6)	0.040	93 (5.9)	27 (5.1)	0.033
Omega-3 fatty acid	407 (3.6)	20 (3.5)	0.009	43 (2.7)	17 (3.2)	0.030

Data are expressed as mean ± SD or *n* (%). Standardized mean difference (SMD) with ≤0.1 was considered well balanced after matching

*Abbreviations*: *ACEi* angiotensin-converting enzyme inhibitor, *ARB* angiotensin receptor blocker, *RAS* renin–angiotensin system

^*^indicating significantly different between the two group

### Primary endpoint: renal composite outcomes

**Fig. 1:**
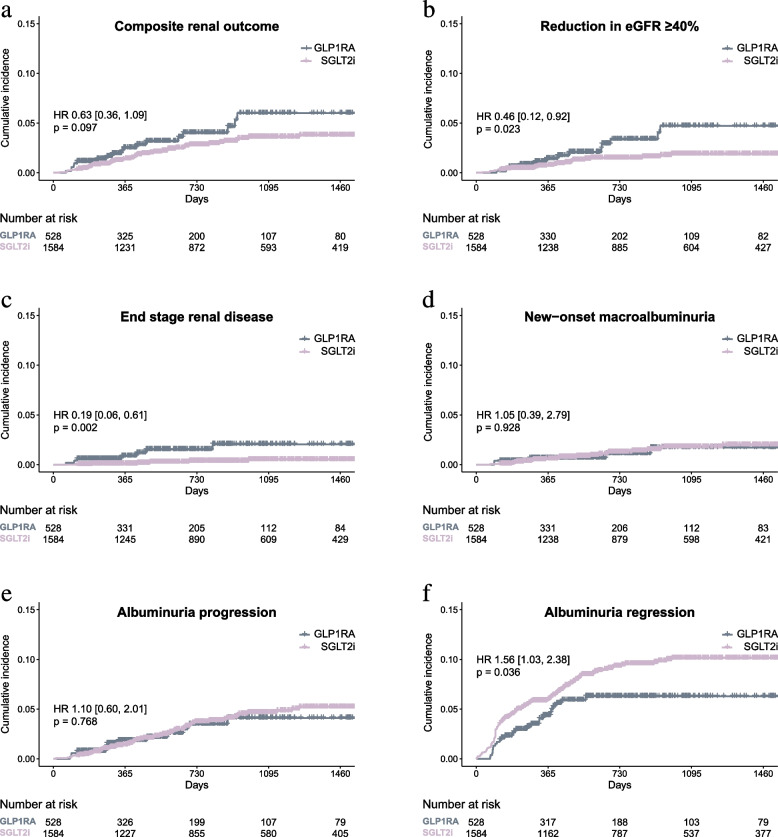
Cumulative incidence of renal outcomes. **a** Composite renal outcomes, (**b**) sustained reduction in eGFR ³40%, (**c**) end-stage renal disease, (**d**) new-onset macroalbuminuria, (**e**) albuminuria progression, and (**f**) albuminuria regression. GLP1RA, glucagon-like peptide-1 receptor agonists; SGLT2i, sodium–glucose co-transporter-2 inhibitors. *p* values were calculated for the log-rank test conducted between the two groups

Table 2 and Fig. 1 detail renal outcomes at a median follow-up of 731 days (interquartile range [IQR], 327–1,408). The primary composite renal outcomes occurred in 18 patients (3.4%) administered a GLP1RA and 42 patients (2.7%) administered an SGLT2i, corresponding to incidence rates of 16.9 and 9.4 per 1,000 person-years, respectively (Table 2), suggesting a trend toward risk reduction for composite renal outcomes with SGLT2i compared with GLP1RA (Fig. 1a). SGLT2i therapy significantly reduced the risk of a sustained eGFR decrease compared with GLP1RA (≥ 40% reduction in eGFR: HR, 0.46; 95% confidence interval [CI], 0.12–0.92) (Fig. 1b) and increased the likelihood of albuminuria regression (HR, 1.56; 95% CI, 1.03–2.38) (Fig. 1f). Subgroup analyses did not reveal any significant interactions stratified by age, sex, baseline eGFR, or albuminuria status (Additional file 1: Table S3).

**Table 2:** Incidence rate of renal outcomes and comparison between GLP1RA and SGLT2i users

	SGLT2i (*n* = 1,584)			GLP1RA (*n* = 528)			SGLT2i vs GLP1RA		
Outcomes	Cumulative incidence (n, %)		Incidence rate (Events/1,000 PY)	Cumulative incidence (n, %)		Incidence rate (Events/1,000 PY)	HR	95% CI	*p*
Composite renal outcome^a^	42	(2.7)	9.4	18	(3.4)	16.9	0.63	0.36, 1.09	0.097
*Its individual component*									
Reduction in eGFR ³40%	22	(1.4)	4.9	13	(2.5)	12.0	0.46	0.12, 0.92	0.023
End-stage renal disease	5	(0.3)	1.1	7	(1.3)	6.4	0.19	0.06, 0.61	0.002
Renal death	0	(0)	–	0	(0)	–	–	–	–
New-onset macroalbuminuria	20	(1.3)	4.5	5	(0.9)	4.6	1.05	0.39, 2.79	0.928
*Other pre-specified outcomes*									
Reduction in eGFR ³50%	9	(0.6)	2.0	8	(1.5)	7.3	0.30	0.12, 0.78	0.013
Doubling of serum creatinine	6	(0.4)	1.3	7	(1.3)	6.4	0.22	0.07, 0.65	0.006
Albuminuria progression	54	(3.4)	12.3	13	(2.5)	12.2	1.10	0.60, 2.01	0.768
Albuminuria regression	133	(8.4)	32.2	26	(4.9)	25.1	1.56	1.03, 2.38	0.036

*Abbreviations*: *HR* hazard ratio, *CI* confidence interval, *PY* people years

^a^The composite renal outcome is defined as a sustained reduction in eGFR ³40%, progression to end-stage renal disease, renal death, or the new-onset of macroalbuminuria

### Changes in renal or metabolic measurements

**Fig. 2:**
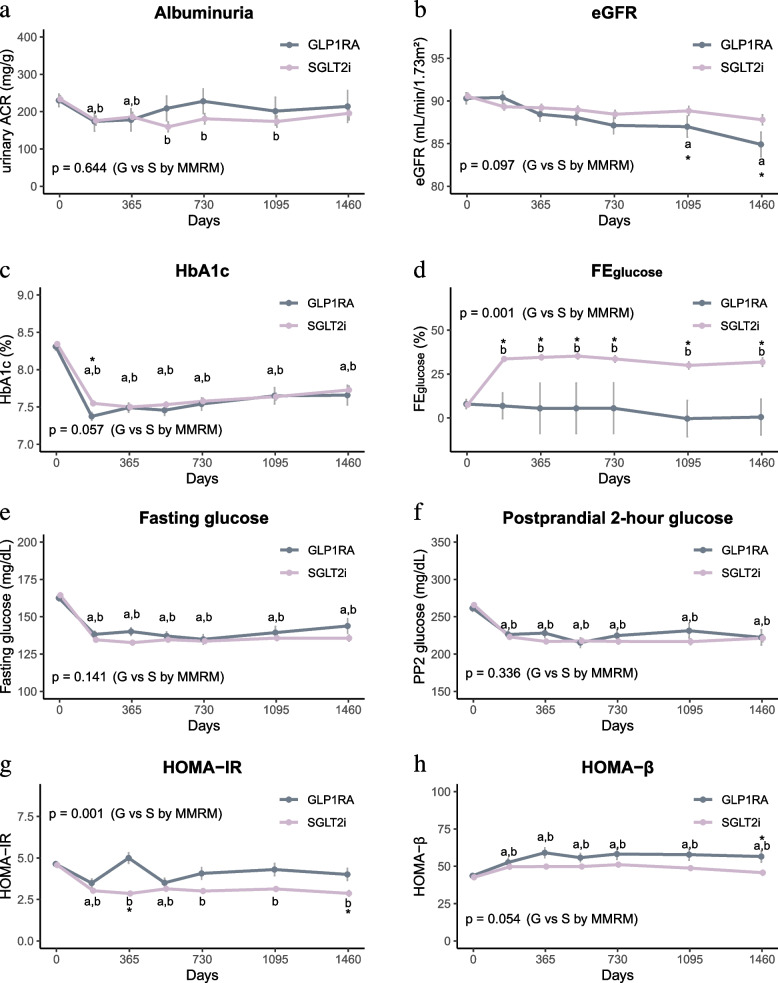
Changes in clinical parameters related to renal function and glycaemic control: (**a**) albuminuria, (**b**) eGFR, (**c**) HbA_1c_, (**d**) FE_glucose_, (**e**) fasting glucose, (**f**) postprandial 2-h glucose, (**g**) HOMA-IR, and (**h**) HOMA-β. ACR, albumin-to-creatinine ratio; FE, fractional excretion. ^a^Significant change from baseline with GLP1RAs. ^b^Significant change from baseline with SGLT2is. ^*^Significant difference between the two groups of changes from the baseline value by paired comparison. *p* values indicate the difference between the two groups by mixed-effect models for repeated measures (MMRM) by defined time point

Figure 2 and Additional file 1: Figs S2–S5 illustrate clinical parameters related to renal function, glycaemic metabolism, lipids, and anthropometry. Throughout the observation period, SGLT2i therapy consistently reduced urinary ACR and preserved eGFR levels (Fig. 2a and b). In contrast, eGFR levels decreased gradually in people with GLP1RA therapy, displaying a trend toward a group difference (*p* = 0.097).

There were no significant differences in HbA_1c_ and glucose levels between groups (Fig. 2c, e, and f). However, a greater proportion of patients reached the target HbA_1c_ level (< 7%) with GLP1RA therapy than with SGLT2i therapy (40.6% vs 31.4%; *p* < 0.001). In the present study, urinary glucose excretions and electrolytes were measured in 60% of patients. SGLT2i therapy was associated with an increase to 33.6% in FE_glc_ at 180 days, which was maintained within a range from 30.0% to 35.1% throughout the observation period (Fig. 2d). This increase was not observed with GLP1RA therapy. FE_Na_ increased with long-term GLP1RA therapy (Additional file 1: Fig. S2a). Overall, SGLT2i therapy decreased HOMA-IR, whereas GLP1RA therapy increased HOMA-β (Fig. 2g and h).

Regarding lipid profiles, GLP1RA therapy was more effective in reducing total cholesterol and LDL-cholesterol (Additional file 1: Fig. S3a and S3d), while SGLT2i therapy was better for controlling blood pressure and body weight (Additional file 1: Fig. S3e–S3h). Ketone levels increased with SGLT2i therapy, although the difference between the two groups was not statistically significant (Additional file 1: Fig. S4). SGLT2i therapy resulted in an increase in muscle percentage and a decrease in body fat percentage; however, no significant group differences were observed (Additional file 1: Fig. S5).

We examined the association between use of RAS blockers on albuminuria and renal function and the outcomes of the therapies (RAS blocker users: *n* = 1,129; RAS blocker nonusers: *n* = 788). Consistent use of RAS blockers tended to be associated with more favourable composite renal outcomes for patients administered a SGLT2i than for patients administered a GLP1RA (HR, 0.52; 95% CI, 0.26–1.00).

When matched further with the year of medication initiation (Additional file 1: Table S4), the results were similar to those from the original cohort and favoured SGLT2i over GLP1RA in terms of renal function deterioration and development of ESRD (significant) and albuminuria progression (trend).

Furthermore, a more advanced baseline kidney stage was correlated with an increased risk of primary composite renal outcomes, as shown in Additional file 1: Table S5. SGLT2is were found to significantly lower the sHR of composite renal outcomes compared to GLP1RAs in analyses utilizing multivariable risk regression models that included albuminuria and eGFR as variables.

### Safety issues

A total of 743 patients (35.3%) reported one or more adverse events (GLP1RA group, *n* = 203 [38.4%]; SGLT2i group, *n* = 543 [34.3%]) (Additional file 1: Table S6). Overall, the incidence of adverse events was comparable between groups. The GLP1RA group experienced nausea and vomiting more frequently than the SGLT2i group (6.4% vs 0.6%; *p* < 0.001). The SGLT2i group tended to experience genitourinary infection more frequently than the GLP1RA group, but the difference was not significant (4.7% vs 3.8%; *p* = 0.394). For cardiovascular events, the HR was calculated (Additional file 1: Table S7) and was not significantly different between the two groups (HR, 1.84; 95% CI, 0.41–8.23). Serious adverse events were not significantly different between the two groups.

## Discussion

In the present study of data from patients with type 2 diabetes, which employed 1:3 propensity-score matching (528 and 1,584 patients with GLP1RA and SGLT2i therapies, respectively), the incidence of primary composite renal outcomes for a ≤ 4-year follow-up were 16.9 and 9.4 per 1,000 person-years, respectively, with no significant difference found between the two agents. SGLT2i therapy significantly reduced the risk of a sustained eGFR decrease in individual components compared with GLP1RA, in either a ≥ 40% or ≥ 50% decrease in eGFR. Patients administered with SGLT2i also displayed a significantly increased likelihood of albuminuria regression than those administered with GLP1RA.

Notably, previous studies have provided mixed evidence concerning the relative effectiveness of SGLT2i and GLP1RA therapies on composite renal outcomes. A network analysis of 15 cardiovascular outcome trials (CVOTs) reported a 22% risk reduction in composite renal outcomes with SGLT2i compared to GLP1RA (relative risk [RR], 0.78; 95% CI, 0.65–0.93) [7]. In our recent network meta-analysis of 43 trials that compared nine types of glucose-lowering therapies [3], GLP1RA and SGLT2i therapies were associated with lower risks for composite renal outcome than placebo (22% and 34%, respectively), but no significant difference between GLP1RA and SGLT2i therapies was noted.

In a recent analysis using the Hong Kong Hospital Authority database involving 5,102 patients, SGLT2i users had a lower risk of composite renal outcomes than GLP1RA users (RR, 0.77; 95% CI, 0.62–0.96), mainly driven by a reduced development of ESRD (HR, 0.53; 95% CI, 0.33–0.86, *p* = 0.01) [20]. The beneficial impact of the use of SGLT2is in mitigating renal function decline observed in our study was not mirrored in the Hong Kong database study [20].

Of note, there are some differences between the two studies in their baseline characteristics. At baseline, the proportion of patients with normal eGFR levels or normoalbuminuria were higher in our cohort than in the Hong Kong study (86.9% vs 71.2% and 57.5% vs 44.1%, respectively). Additionally, we adopted a ≥ 40% eGFR reduction as the standard criterion for renal impairment, but the Hong Kong study adopted a ≥ 50% eGFR reduction.

In the most recent findings, the FLOW trial reported a 24% reduction in the progression of kidney disease and mortality associated with the administration of semaglutide 1.0 mg [21, 22]. It is crucial to acknowledge, however, that this trial was placebo-controlled and employed a GLP1RA, rather than an SGLT2i, in participants with type 2 diabetes and chronic kidney disease. These results underscore the necessity for direct comparative studies to more accurately determine the relative efficacy and safety of these treatments.

The underlying mechanisms by which SGLT2is and GLP1RAs manage glucose demonstrate clear distinctions. SGLT2i treatment improves glucose regulation primarily by augmenting glycosuria and attenuating insulin resistance [23]. By contrast, GLP1RAs mainly act on pancreatic β-cell function, systemic inflammation, and satiety neurons in the hypothalamus in the central nervous system [24]. While overall glucose regulation did not show significant disparities between the two therapeutic modalities, a more substantial fraction of patients treated with GLP1RA reached HbA_1c_ values < 7% in the present study. This finding could be attributed to the fundamental pharmacological features of GLP1RA to enhance β-cell function and alleviate insulin resistance.

In the present study, GLP1RA therapy decreased total and LDL-cholesterol levels. It was reported that GLP1RA therapy is able to modulate lipogenesis and β-oxidation in liver [25] and to improve insulin sensitivity by promoting the degradation of apolipoprotein-B through phosphatidylinositol 3-kinase [26]. By contrast, it is noted that the reported impact of SGLT2is on cholesterol levels has not been consistent. A meta-analysis of 48 RCTs including 24,782 participants revealed increases in LDL- and HDL-cholesterol levels with SGLT2i therapy by 3.89 mg/dL (95% CI, 0.07–0.12) and by 2.33 mg/dL (95% CI, 0.05–0.08), respectively, compared to the control group [27]. Of note, the increase in HDL-cholesterol were more pronounced in Asians [27]. In the present study, SGLT2i therapy led to an increase in HDL-cholesterol, but a decrease in LDL-cholesterol. A recent meta-analysis reported that SGLT2i therapy increased LDL-cholesterol, but this effect was not observed with 10 mg of empagliflozin or dapagliflozin [28]. More than 95% of the cases in our study used these drugs at the same doses. Considering the lack of significant changes in lipid-lowering therapy in our study, the substantial improvement in insulin resistance and significant reduction in body weight by SGLT2i treatment might be attributable to LDL-cholesterol reduction [29].

In this study, the likelihood of achieving the target blood pressure was two-fold higher with SGLT2i therapy than with GLP1RA therapy. This result is in line with a previous study of Japanese subjects with type 2 diabetes [30].

We found that body weight was reduced more markedly with SGLT2i therapy than with GLP1RA therapy. In the large-scale RCTs with the medications used in our cohort, there was slightly greater weight loss with SGLT2i than GLP1RA (–2 kg for SGLT2i vs –1.5 kg for GLP1RA) (Additional file 1: Table S8) [9, 31–36]. Japanese studies showed similar findings [30, 37]. Notably, the GLP1RAs included in the current analysis were used at doses for diabetes management. Whereas GLP1RAs at higher doses were very effective for obesity management [38, 39]. Favourable effects of SGLT2i for lowering glucose [40] and heart failure and cardiovascular death were found more prominent in those with South Asian and East/Southeast Asian ancestry (defined as Asians) than in those with Western European ancestry in a meta-analysis [41]. The relatively lower body mass and high salt and high carbohydrate diet of Asian populations may be associated with these results [42].

In the present study, SGLT2i therapy increased ketone levels throughout the observation period, whereas GLP1RA therapy did not, which is consistent with previous studies [43, 44]. Ketone bodies are an ancillary fuel source substituting for glucose in the heart, and exhibit antioxidative and anti-inflammatory effects [45]. Ketone bodies require less oxygen to produce the same amount of energy than glucose, potentially improving organ function under conditions of stress [45]. An alteration of energy source by SGLT2i therapy can be linked to several advantages, including enhanced mitochondrial biogenesis and function, effective energy utilization, and increased erythropoiesis, all contributing to renal benefits [46].

In this study, there were no significant differences in overall adverse events between the two treatment groups. As anticipated, GLP1RA therapy was associated with gastrointestinal disturbances, including nausea and vomiting. SGLT2i therapy is typically linked with genital tract infections, but this was not observed in our present analysis, which might be due to the strict education regarding subjects maintaining hydration and good hygiene. In a recent large study (EMPA-KIDNEY), the use of empagliflozin did not increase the incidence of serious urinary tract infections, acute kidney injuries, symptomatic dehydration, or bone fractures [12].

Intriguingly, in the sensitivity analysis, the renoprotective effects of SGLT2i therapy were more pronounced with the simultaneous use of a RAS blocker. The renovascular benefits of RAS blockers for individuals with renal disease are well documented [47]. The combined use of a SGLT2i and RAS blocker curtails oxidative stress, as demonstrated by a decrease in the 8-isoprostane marker [48]. The recuperated tubuloglomerular feedback mechanisms are also associated with a decrease in the risk of cardiorenal complications [48].

### Strengths and limitations

The present study has several distinctive features. First, 20.7% of the study participants had a prevalence of pre-existing CVDs, which was lower than previous CVOTs ranging from 31.5–100% [5]. This result enhanced the possible insights. Second, unique to the present study were the comparisons between the two drug classes for specific in-hospital tests, such as FE_glc_, FE_Na_, FE_K_, ketone levels, and body composition, which are not available in other large database studies [20]. Third, the observation period was relatively prolonged, with a median of 731 days (IQR, 327–1,408 days). However, there are several caveats for our analysis.

The first caveat is that the findings might not apply to other populations in the same way, as our data were derived from a cohort of East Asian ancestry. Second, the patients whose data were included in this study had a higher prevalence of diabetic kidney disease than adults with diabetes overall from Korean nationwide data (i.e. 36.6% vs 27.6%) [49]. However, the characteristics of the patients whose data were included in our study are similar to those of most patients with diabetes attending referral hospitals [50]. When we compared unmatched SGLT2i subjects with matched SGLT2i subjects, most characteristics were similar, except for slightly higher numbers of comorbidities and a lower BMI in the unmatched group. This difference means that SGLT2is were more prescribed for patients with more complications, particularly heart failure, whereas GLP1RAs were more prescribed for those with obesity. Third, we primarily investigated the class effects of SGLT2is and GLP1RAs, rather than the individual agents within the class.

## Conclusions

In summary, SGLT2i therapy may offer more distinctive benefits in renal protection, particularly in the reduction in albuminuria and mitigation in eGFR decline, compared with GLP1RAs at doses prescribed for diabetes management. In contrast, GLP1RAs may emerge as a favourable choice for glucose regulation and dyslipidaemia management. Future head-to-head studies comparing the two agents or including recent potent GLP1RAs and GLP1/GIP co-agonists, to expand on the present observations, and thereby enhance tailored therapeutic approaches for managing type 2 diabetes are warranted.

### Supplementary Information

Additional file 1: Table S1. Definition of outcomes and diseases. Table S2. Types of SGLT2i or GLP1RA used in each cohort. Table S3. Incidence rate of composite renal outcome and its comparison between GLP1RA and SGLT2i users according to baseline characteristics. Table S4. Incidence rate of renal outcomes and comparison between GLP1RA and SGLT2i users in patients further matched with the year of medication start. Table S5. Multivariable competing risk regression estimates of subdistribution hazard ratio for the composite renal outcome with Fine and Gray Model. Table S6. Adverse events. Table S7. Incidence rate of cardiovascular outcomes and comparison between GLP1RA and SGLT2i users. Table S8. Weight changes after GLP1 receptor agonists and SGLT2 inhibitors therapy in major randomized controlled studies. Fig. S1. Study design flow chart. Fig. S2. Changes in clinical parameters related to renal function: (a) FE_Na_, and (b) FE_K_. Fig. S3. Changes in clinical parameters of metabolic parameters: (a) total cholesterol, (b) triglycerides, (c) HDL-cholesterol, (d) LDL-cholesterol, (e) SBP, (f) DBP, (g, i) body weight, and (h, j) BMI. Fig. S4. Changes in glucagon and ketone bodies: (a) glucagon, (b) total ketone, (c) β-hydroxybutyrate, and (d) acetoacetate. Fig. S5. Changes in body composition: (a) whole body muscle mass, (b) whole body fat mass, (c) muscle percentage, and (d) fat percentage.

## Data Availability

The datasets generated during and/or analysed during the current study are available from the corresponding author on reasonable request.

## Abbreviations

ACEi: Angiotensin-converting enzyme inhibitor
ACR: Albumin-to-creatinine ratio
ARB: Angiotensin II receptor blocker
BMI: Body mass index
CVD: Cardiovascular disease
CVOT: Cardiovascular outcome trial
DBP: Diastolic blood pressure
eGFR: Estimated glomerular filtration ratio
ESRD: End-stage renal disease
FEglc: Fraction excretion of glucose
FEK: Fraction excretion of potassium
FENa: Fraction excretion of sodium
GLP1RA: Glucagon-like peptide-1 receptor agonists
HbA1c: Glycated haemoglobin
HDL-c: High-density lipoprotein cholesterol
HOMA-IR: Homeostatic model assessment for insulin resistance
HOMA-β: Homeostatic model assessment for β-cell function
HR: Hazard ratio
LDL-c: Low-density lipoprotein cholesterol
PP2: Postprandial 2-h
RAS: Renin–angiotensin system
RCT: Randomized controlled trial
SBP: Systolic blood pressure
SGLT2i: Sodium–glucose co-transporter-2 inhibitors
sHR: Subdistribution hazard ratios
SMD: Standardized mean difference
uACR: Urinary albumin-creatinine-ratio
