# Supplementary material for: Long-term comparison of renal and metabolic outcomes after sodium–glucose co-transporter 2 inhibitor or glucagon-like peptide-1 receptor agonist therapy in type 2 diabetes
Source: BMC Med. 2024 Jul 2;22:273. doi: 10.1186/s12916-024-03483-z (PMC11218058; doi:10.1186/s12916-024-03483-z)

Additional file 1 of 'Long-term comparison of renal and metabolic outcomes after sodium–glucose co-transporter 2 inhibitor or glucagon-like peptide-1 receptor agonist therapy in type 2 diabetes'

Table S1. Definition of outcomes and diseases

Outcome or disease	Definition
Composite renal outcomes	The components of the renal composite endpoints, including sustained decrease in eGFR, confirmed end-stage renal disease, renal death, or new-onset macroalbuminuria
Sustained reduction in eGFR	Sustained requires two consecutive measures that meet the requirement and are at least 30 days apart
End-stage renal disease	Sustained eGFR of <15 mL/min/1.73 m^2, or requirement of renal replacement therapy including chronic dialysis or transplantation
New-onset macroalbuminuria	The development of macroalbuminuria in a subject with baseline state of normoalbuminuria or microalbuminuria that are confirmed consecutively at least twice, with change of albuminuria at least 20% and each confirmation being at least 30 days apart
Albuminuria progression	The development of macroalbuminuria in a subject with baseline state of normoalbuminuria or microalbuminuria, or development of microalbuminuria in a subject with baseline state of normoalbuminuria that are confirmed consecutively at least twice, with change of albuminuria at least 20% and each confirmation being at least 30 days apart. For last observations, increases in two stages were also included.
Albuminuria regression	The recovery to normoalbuminuria in a subject with baseline state of microalbuminuria or macroalbuminuria, or the improvement to microalbuminuria in a subject with baseline state of macroalbuminuria that are confirmed consecutively at least twice, with change of albuminuria at least 20% and each confirmation being at least 30 days apart. For last observations, decreases in two stages were also included.

Table S2. Types of SGLT2i or GLP1RA used in each cohort

Types of medication	n	%
SGLT2i group		
Dapagliflozin	803	50.7
Empagliflozin	724	45.7
Ipragliflozin	31	2.0
Ertugliflozin	22	1.4
Enavogliflozin	4	0.3
GLP1RA group		
Dulaglutide	346	65.5
Liraglutide*	138	26.1
Exenatide	22	4.2
Lixisenatide	22	4.2

*Diabetic dosages were used.

Table S3. Incidence rate of composite renal outcome and its comparison between GLP1RA and SGLT2i users according to baseline characteristics

	SGLT2i (n = 1,584) Cumulative incidence			GLP1RA (n = 528) Cumulative incidence			SGLT2i vs GLP1RA			
	n	New events	Rate	n	New events	Rate	HR	95% CI	*p* value	*p* for interaction
Overall	1,584	42	2.7%	528	18	3.4%	0.63	0.36–1.09	0.097	–
Sex										
Men	889	23	2.6%	279	9	3.2%	0.66	0.31–1.44	0.297	0.866
Women	695	19	2.7%	249	9	3.6%	0.60	0.27–1.34	0.214	
Age										
Age ≥ 65 years	438	17	3.9%	131	6	4.6%	0.75	0.30–1.90	0.545	0.598
Age < 65 years	1,146	25	2.2%	397	12	3.0%	0.55	0.28–1.11	0.089	
Kidney function										
eGFR–MDRD ≥ 60	1,382	26	1.9%	453	8	1.8%	0.84	0.38–1.86	0.664	0.421
eGFR–MDRD < 60	202	16	7.9%	75	10	13.3%	0.53	0.24–1.17	0.118	
Albuminuria										
Urinary ACR ≥30 mg/g	669	37	5.5%	228	15	6.6%	0.69	0.38–1.25	0.219	0.477
Urinary ACR <30 mg/g	915	5	0.5%	300	3	1.0%	0.39	0.09–1.65	0.201	

Abbreviations: ACR, albumin-creatinine ratio; HR, hazard ratio; CI, confidence interval. The composite renal outcome is defined as a sustained reduction in eGFR ≥40%, progression to end-stage renal disease, renal death, or the new-onset of macroalbuminuria.

Table S4. Incidence rate of renal outcomes and comparison between GLP1RA and SGLT2i users in patients further matched with the year of medication start

Outcomes	SGLT2i (n = 370)		GLP1RA (n = 370)		SGLT2i vs GLP1RA		
	Cumulative incidence (n, %)	Incidence rate (Events/1,000 PY)	Cumulative incidence (n, %)	Incidence rate (Events/1,000 PY)	HR	95% CI	*p*
Composite renal outcome[†]	10 (2.7)	9.0	17 (4.6)	23.8	0.45	0.21, 0.99	0.044
Its individual component							
Reduction in eGFR ≥40%	8 (2.2)	7.2	14 (3.8)	19.3	0.42	0.17, 0.99	0.044
End-stage renal disease	3 (0.8)	2.7	10 (2.7)	13.7	0.14	0.11, 0.17	0.001
Renal death	0 (0)	–	0 (0)	–	–	–	–
New-onset macroalbuminuria	4 (1.1)	3.6	7 (1.9)	9.7	0.43	0.12, 1.46	0.165
Other pre-specified outcomes							
Reduction in eGFR ≥50%	5 (1.4)	4.5	11 (3.0)	15.0	0.34	0.12, 0.97	0.035
Doubling of serum creatinine	1 (0.3)	0.9	7 (1.9)	9.5	0.10	0.01, 0.83	0.007
Albuminuria progression	4 (1.1)	3.6	9 (2.4)	12.5	0.34	0.10, 1.09	0.057
Albuminuria regression	16 (4.3)	14.9	21 (5.7)	25.0	1.49	0.78, 2.87	0.222

Abbreviations: HR, hazard ratio; CI, confidence interval; PY, people years. [†]The composite renal outcome is defined as a sustained reduction in eGFR ≥40%, progression to end-stage renal disease, renal death, or the new-onset of macroalbuminuria.

Participants were matched based on age, sex, BMI, SBP or hypertension, dyslipidaemia, background antidiabetic medications, and the year of medication initiation, with an average start year of 2018 for both groups.

Table S5. Multivariable competing risk regression estimates of subdistribution hazard ratio (sHR) for the composite renal outcome with Fine and Gray Model

Parameter	Parameter estimate	sHR	*p* value
Albuminuria category†			
A1 Normoalbuminuria (urinary ACR < 30 mg/g)	Reference	Reference	–
A2 Microalbuminuria (30 mg/g ≤ urinary ACR < 300 mg/g)	2.01	7.47 (4.26 to 13.1)	< 0.001
A3 Macroalbuminuria (urinary ACR ≥ 300 mg/g)	1.80	6.02 (3.17 to 11.4)	< 0.001
eGFR (mL/min/1.72 m^2) category†			
G1 (eGFR ≥ 90)	Reference	Reference	–
G2 (60 ≤ eGFR < 90)	0.76	2.14 (1.31 to 3.48)	0.007
G3 (30 ≤ eGFR < 60)	1.64	5.18 (3.06 to 8.78)	< 0.001
G4 (15 ≤ eGFR < 30)	3.21	24.9 (11.8 to 52.4)	< 0.001
Intervention group‡			
GLP1RA group	Reference	Reference	–
SGLT2i group	–0.50	0.60 (0.40 to 0.93)	0.020

Abbreviations: eGFR, estimated glomerular filtration ratio; ACR, urinary albumin-creatinine ratio. The composite renal outcome is defined as a sustained reduction in eGFR ≥40%, progression to end-stage renal disease, renal death, or the new-onset of macroalbuminuria.

† The prognosis of kidney disease was stratified by albuminuria and eGFR categories, as defined by the Kidney Disease: Improving Global Outcomes (KDIGO) CKD Work Group.

‡ The multivariable model was stratified by the albuminuria and eGFR categories, as well as by the intervention group, as provided in the table.

Table S6. Adverse events

	SGLT2i (n = 1,584)	GLP1RA (n = 528)	*p* value
Any adverse events	540 (34.1%)	203 (38.4%)	0.093
Serious adverse event	207 (13.1%)	55 (10.4%)	0.109
Hypoglycaemia	145 (9.2%)	62 (11.7%)	0.083
Severe hypoglycaemia	2 (0.1%)	2 (0.4%)	0.248
Gastrointestinal events	35 (2.2%)	42 (8.0%)	0.001
Nausea, vomiting	9 (0.6%)	34 (6.4%)	0.001
Constipation	18 (1.1%)	6 (1.1%)	1.000
Others (including colitis, etc.)	8 (0.5%)	2 (0.4%)	0.796
Genitourinary events	79 (5.0%)	20 (3.8%)	0.259
Symptomatic infection	8 (0.5%)	2 (0.4%)	0.714
Asymptomatic infection	68 (4.3%)	18 (3.4%)	0.374
Urinary incontinence	3 (0.2%)	0 (0.0%)	0.317
Cardiovascular events	56 (3.5%)	15 (2.8%)	0.443
Ketoacidosis	0 (0.0%)	0 (0.0%)	–
Bone fracture	9 (0.6%)	6 (1.1%)	0.178
Malignancy	8 (0.5%)	2 (0.4%)	0.714

Adverse events that were commonly reported were collected throughout the observation period, including the 90 days after discontinuing SGLT2i or GLP1RA.

Table S7. Incidence rate of cardiovascular outcomes and comparison between GLP1RA and SGLT2i users

Outcomes	SGLT2i (n = 1,584) Cumulative incidence (n, %)	SGLT2i (n = 1,584) Incidence rate (Events/1,000 PY)	GLP1RA (n = 528) Cumulative incidence (n, %)	GLP1RA (n = 528) Incidence rate (Events/1,000 PY)	SGLT2i vs GLP1RA HR	SGLT2i vs GLP1RA 95% CI	SGLT2i vs GLP1RA *p*
Composite cardiovascular outcome[1]	56 (3.5)	4.0	15 (2.8)	2.3	1.74	0.41, 3.23	0.425
Myocardial infarction	36 (2.3)	1.9	10 (1.9)	2.2	0.73	0.24, 3.84	0.724
Stroke	13 (0.8)	0.7	3 (0.6)	1.2	0.56	0.15, 5.37	0.663
Heart failure	10 (0.6)	0.7	2 (0.4)	1.1	0.67	0.06, 7.41	0.751
Peripheral arterial disease leading to limb amputation	1 (0.1)	0.4	1 (0.2)	1.1	0.34	0.02, 5.36	0.440

Abbreviations: HR, hazard ratio; CI, confidence interval; PY, people years. [1]The composite cardiovascular outcome is defined as a myocardial infraction, stroke, and heart failure.

Table S8. Weight changes after GLP1 receptor agonists and SGLT2 inhibitors therapy in major randomized controlled studies.

Medication	Weight change vs placebo	Study duration
**GLP1 receptor agonists**		
Dulaglutide [REWIND trial] [Ref.1]	–1.46 kg	5 years
Liraglutide [LEADER trial] [Ref.2]	–2.3 kg	5 years
Exenatide [EXSCEL trial] [Ref.3]	–1.27 kg	5 years
Lixisenatide [ELIXA trial] [Ref.4]	~ –1 kg	3 years
**SGLT2 inhibitors**		
Dapagliflozin [DECLARE-TIMI 58 trial] [Ref.5]	–2.2 kg	2 years
Empagliflozin [EMPA-REG OUTCOME trial] [Ref.6]	~ –1 kg	4 years
Ertugliflozin [VERTIS-CV trial] [Ref.7]	–2.4 ~ –2.8 kg	4 years

Ref 1. Gerstein HC, *et al*. Lancet 2019, 394(10193):131-138. Ref 2. Marso SP, *et al*. N Engl J Med 2016, 375(4):311-322. Ref 3. Holman RR, *et al*. N Engl J Med 2017, 377(13):1228-1239. Ref 4. Pfeffer MA, *et al*. N Engl J Med 2015, 373(23):2247-2257. Ref 5. Wiviott SD, *et al*. N Engl J Med 2019, 380(4):347-357. Ref 6. Zinman B, *et al*. N Engl J Med 2015, 373(22):2117-2128. Ref 7. Cannon CP, *et al*. N Engl J Med 2020, 383(15):1425-1435.

Fig. S1. Study design flow chart

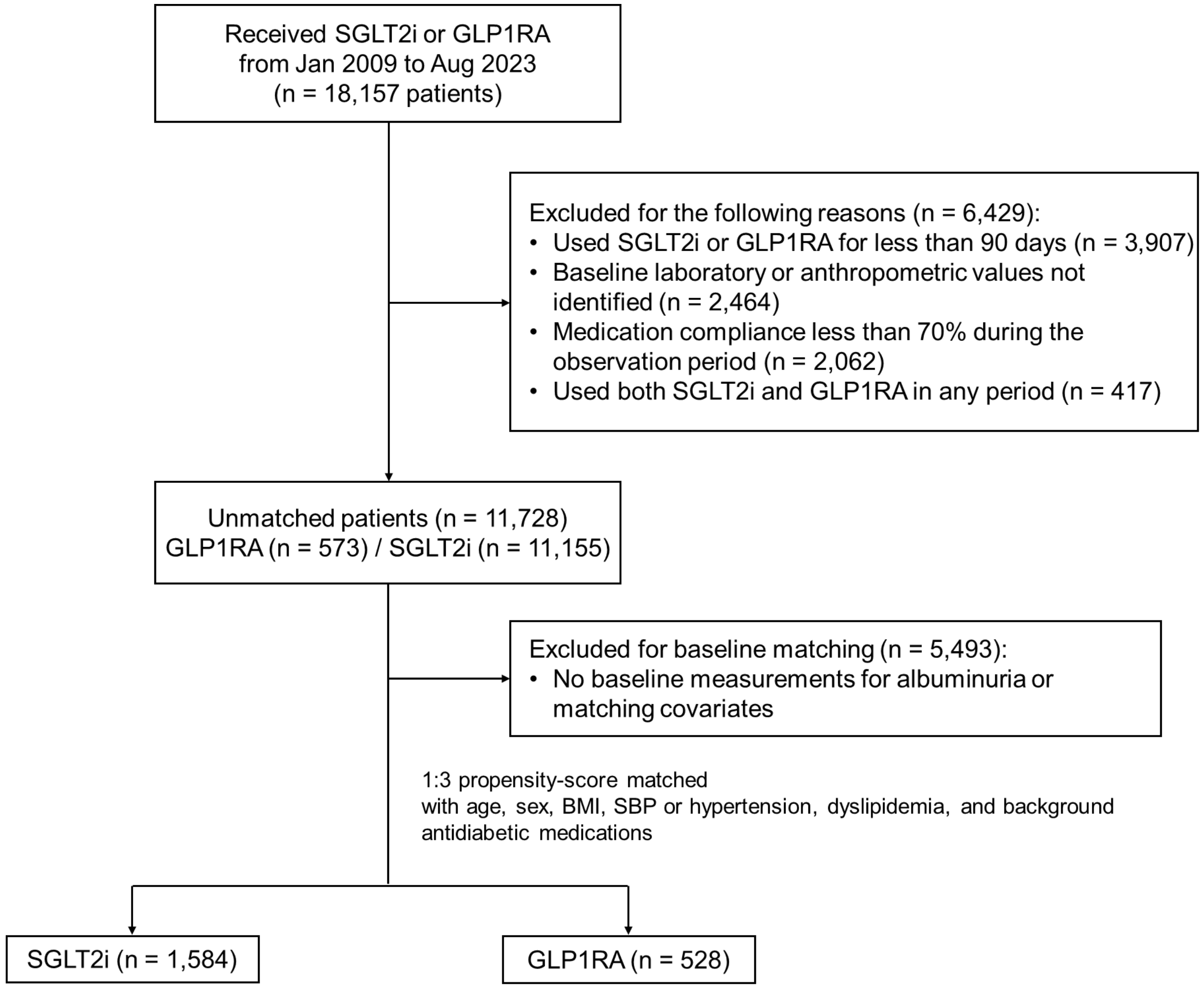

Fig. S2. Changes in clinical parameters related to renal function: (a) FE_{Na}, and (b) FE_{K}. Abbreviations: FE_{K}, fraction excretion of potassium; FE_{Na}, fraction excretion of sodium. [a]Significant change from baseline with GLP1RAs. [b]Significant change from baseline with SGLT2is. [*]Significant difference between the two groups of changes from the baseline value by paired comparison. *p* values on the left show the difference between the two groups by MMRM by defined time point. Data from the patients who reached end-stage renal disease were not included in the current figure, which most affected the FE_{Na} values as expected.

a b

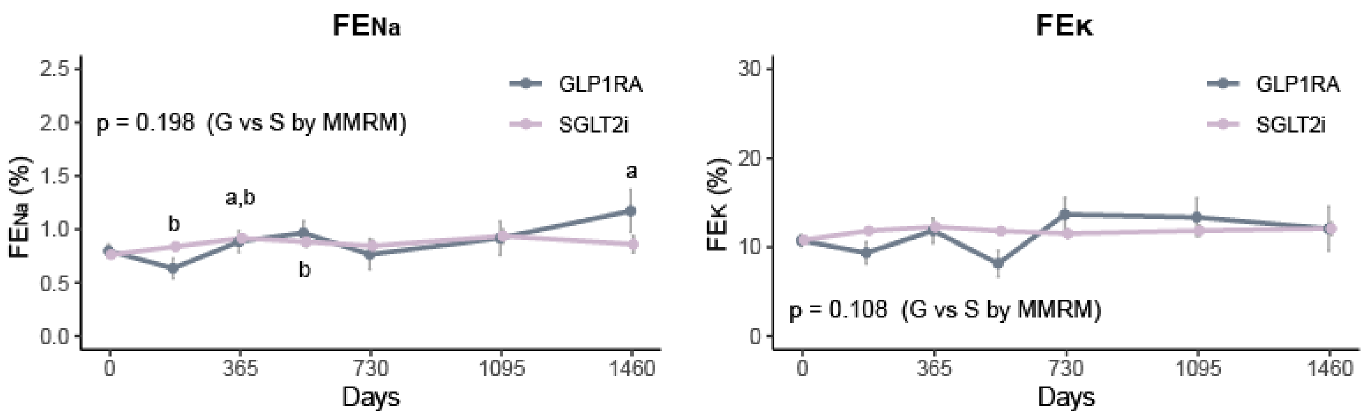

Fig. S3. Changes in clinical parameters of metabolic parameters: (a) total cholesterol, (b) triglycerides, (c) HDL-cholesterol, (d) LDL-cholesterol, (e) SBP, (f) DBP, (g, i) body weight, and (h, j) BMI. [a]Significant change from baseline with GLP1RAs. [b]Significant change from baseline with SGLT2is. [*]Significant difference between the two groups of changes from the baseline value by paired comparison. *p* values on the left show the difference between the two groups by MMRM by defined time point.

a

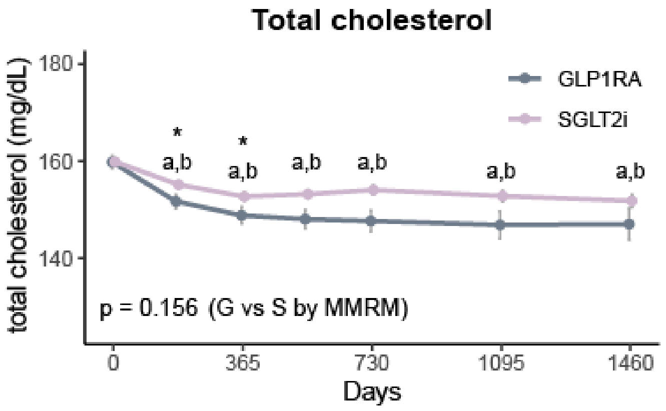

b

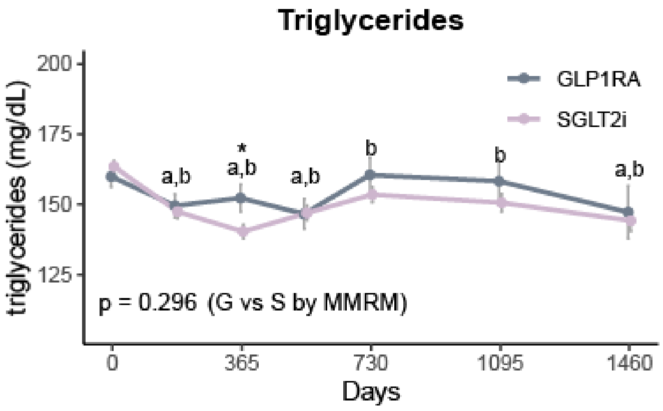

c

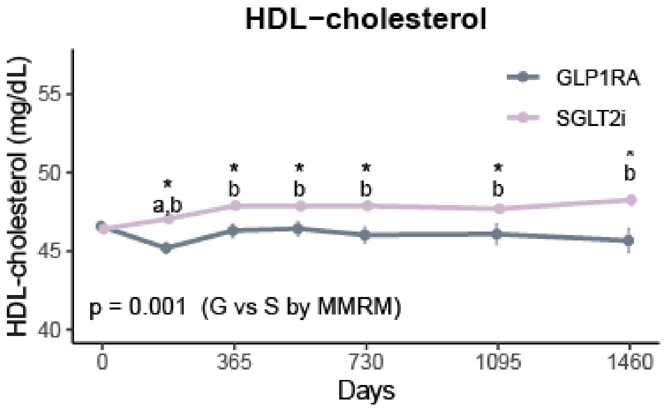

d

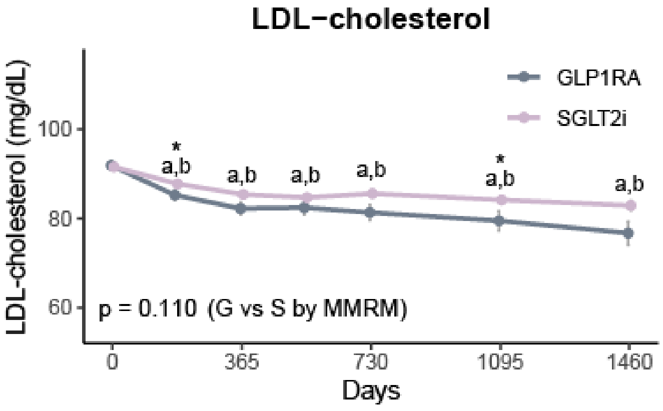

e

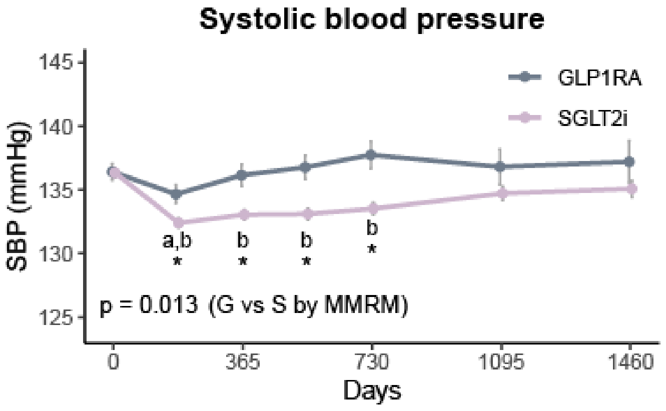
Systolic blood pressure
GLP1RA
SGLT2i
SBP (mmHg)
145
140
135
130
125
a,b
b
b
b
p = 0.013 (G vs S by MMRM)
0
365
730
1095
1460
Days

f

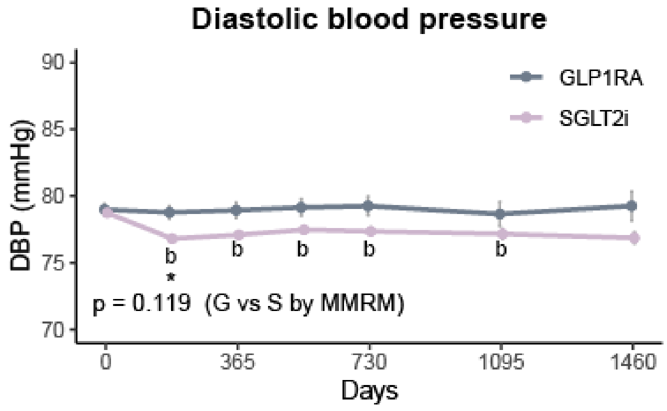
Diastolic blood pressure
GLP1RA
SGLT2i
DBP (mmHg)
90
85
80
75
70
b
b
b
b
b
p = 0.119 (G vs S by MMRM)
0
365
730
1095
1460
Days

g

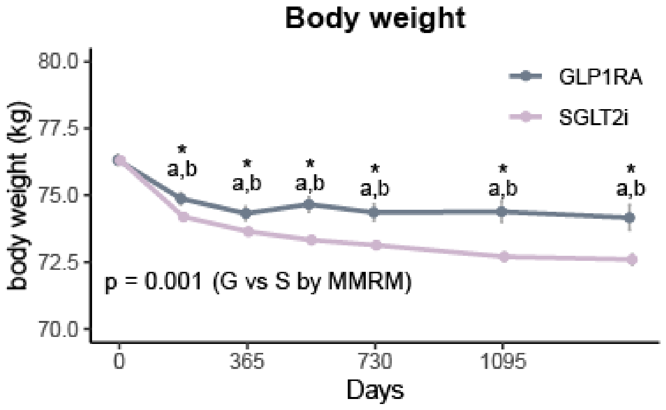
Body weight
GLP1RA
SGLT2i
body weight (kg)
80.0
77.5
75.0
72.5
70.0
a,b
a,b
a,b
a,b
a,b
a,b
p = 0.001 (G vs S by MMRM)
0
365
730
1095
Days

h

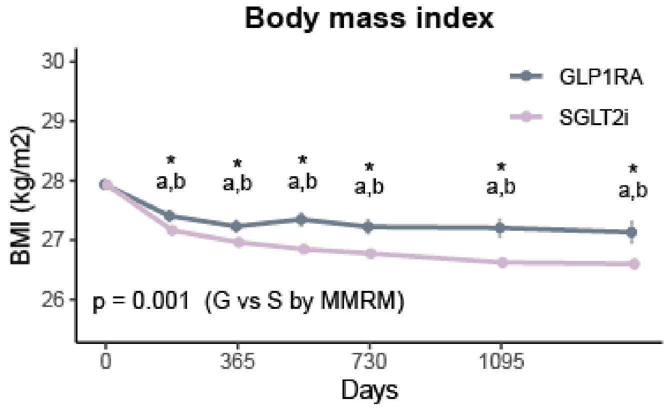
Body mass index
GLP1RA
SGLT2i
BMI (kg/m2)
30
29
28
27
26
a,b
a,b
a,b
a,b
a,b
a,b
p = 0.001 (G vs S by MMRM)
0
365
730
1095
Days

i

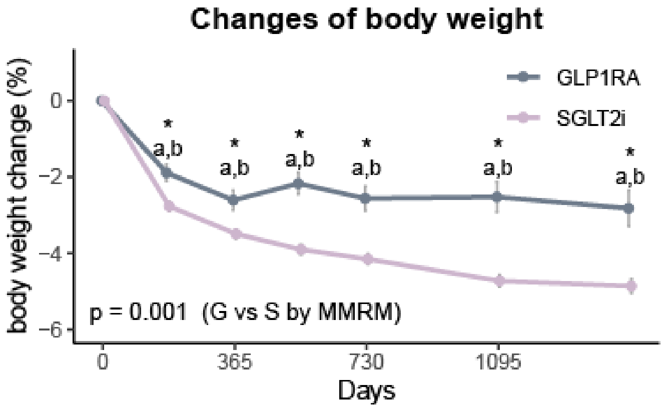
Changes of body weight
GLP1RA
SGLT2i
body weight change (%)
0
−2
−4
−6
a,b
a,b
a,b
a,b
a,b
a,b
p = 0.001 (G vs S by MMRM)
0
365
730
1095
Days

j

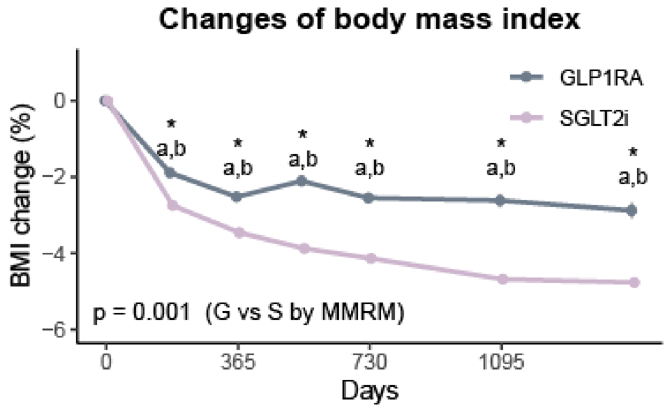
Changes of body mass index
GLP1RA
SGLT2i
BMI change (%)
0
−2
−4
−6
a,b
a,b
a,b
a,b
a,b
a,b
p = 0.001 (G vs S by MMRM)
0
365
730
1095
Days

Fig. S4. Changes in glucagon and ketone bodies: (a) glucagon, (b) total ketone, (c) β-hydroxybutyrate, and (d) acetoacetate. [b]Significant change from baseline with SGLT2is. *p* values on the left indicate the difference between the two groups by MMRM by defined time point. Missing values were imputed using the last observation carried forward method, and log-transformed values were used for comparative analyses due to the substantial variability of the values.

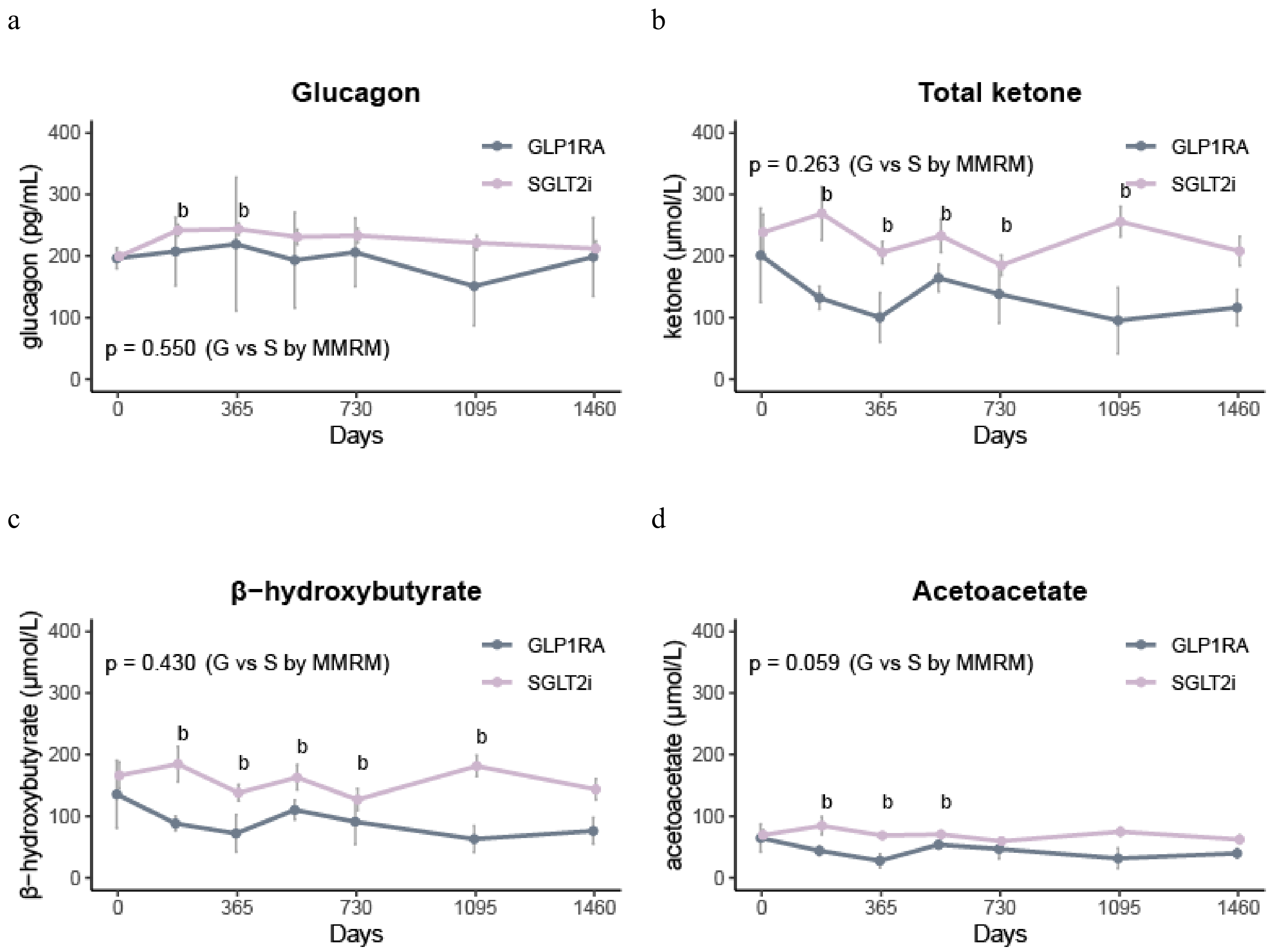

Fig. S5. Changes in body composition: (a) whole body muscle mass, (b) whole body fat mass, (c) muscle percentage, and (d) fat percentage. [a]Significant change from baseline with GLP1RAs. [b]Significant change from baseline with SGLT2is. [*]Significant difference between the two groups of changes from the baseline value by paired comparison. The values before and after treatment were compared between the GLP1RA group (n = 83) and SGLT2i group (n = 268) after averaging the test dates (at 212 days vs 280 days).

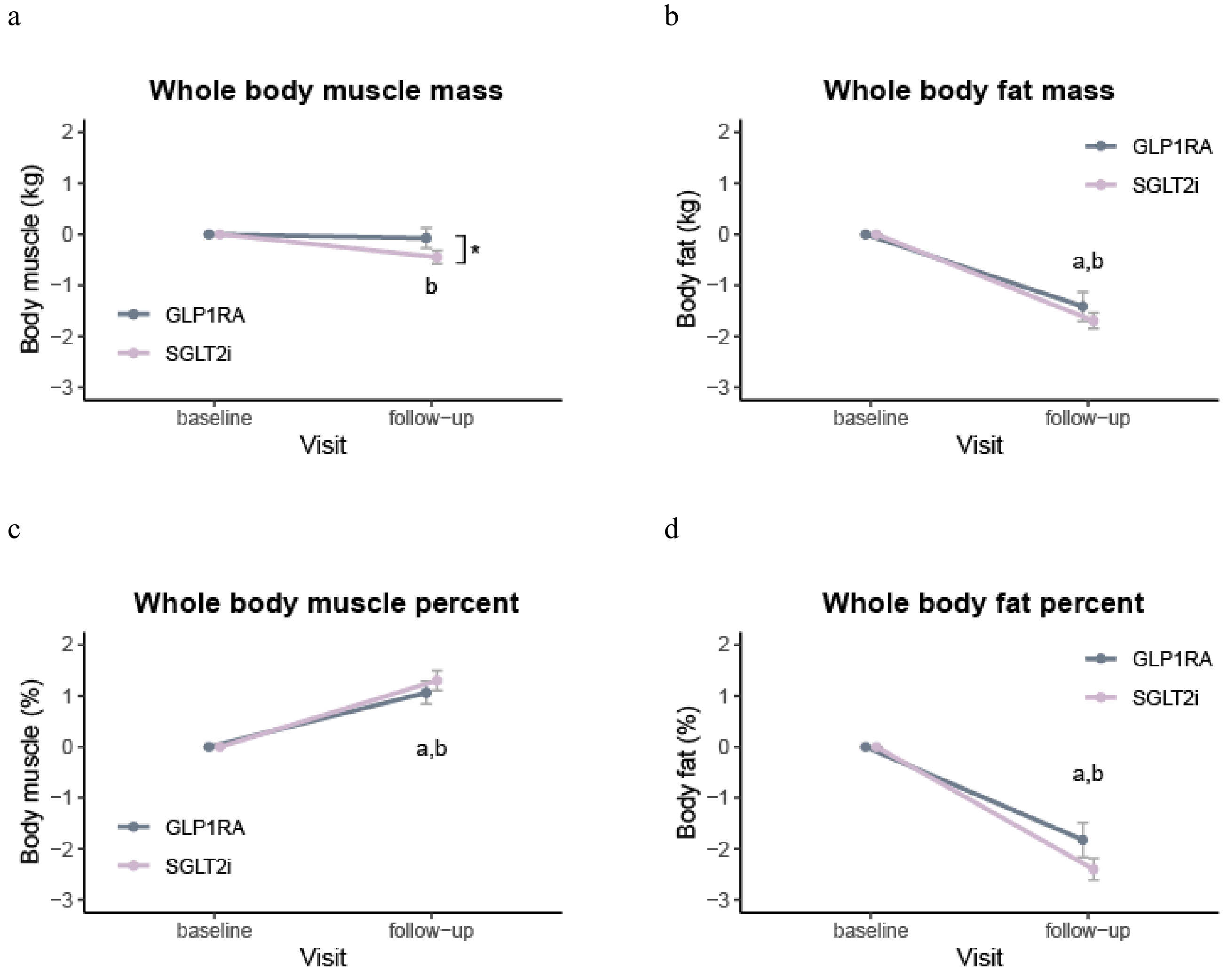

Supplement: Supplementary file 1 — Additional file 1: Table S1. Definition of outcomes and diseases. Table S2. Types of SGLT2i or GLP1RA used in each cohort. Table S3. Incidence rate of composite renal outcome and its comparison between GLP1RA and SGLT2i users according to baseline characteristics. Table S4. Incidence rate of renal outcomes and comparison between GLP1RA and SGLT2i users in patients further matched with the year of medication start. Table S5. Multivariable competing risk regression estimates of subdistribution hazard ratio for the composite renal outcome with Fine and Gray Model. Table S6. Adverse events. Table S7. Incidence rate of cardiovascular outcomes and comparison between GLP1RA and SGLT2i users. Table S8. Weight changes after GLP1 receptor agonists and SGLT2 inhibitors therapy in major randomized controlled studies. Fig. S1. Study design flow chart. Fig. S2. Changes in clinical parameters related to renal function: (a) FENa, and (b) FEK. Fig. S3. Changes in clinical parameters of metabolic parameters: (a) total cholesterol, (b) triglycerides, (c) HDL-cholesterol, (d) LDL-cholesterol, (e) SBP, (f) DBP, (g, i) body weight, and (h, j) BMI. Fig. S4. Changes in glucagon and ketone bodies: (a) glucagon, (b) total ketone, (c) β-hydroxybutyrate, and (d) acetoacetate. Fig. S5. Changes in body composition: (a) whole body muscle mass, (b) whole body fat mass, (c) muscle percentage, and (d) fat percentage. [file 12916_2024_3483_MOESM1_ESM.pdf]
